# ARD1/NAA10 in hepatocellular carcinoma: pathways and clinical implications

**DOI:** 10.1038/s12276-018-0106-1

**Published:** 2018-07-27

**Authors:** Danbi Lee, Myoung-Kuk Jang, Ji Hae Seo, Soo Hyung Ryu, Jeong A. Kim, Young-Hwa Chung

**Affiliations:** 1 Department of Internal Medicine, University of Ulsan College of Medicine, Asan Medical Center, Seoul, Republic of Korea; 2 Department of Internal Medicine, Hallym University College of Medicine, Kangdong Sacred Heart Hospital, Seoul, Republic of Korea; 3 Department of Biochemistry, Keimyung University School of Medicine, Daegu, Republic of Korea; 4 Department of Internal Medicine, Inje University College of Medicine, Seoul Paik Hospital, Seoul, Republic of Korea; 5 DNA Link Inc., Seoul, Republic of Korea

**Keywords:** Protein folding, Cancer

## Abstract

Hepatocellular carcinoma (HCC), a representative example of a malignancy with a poor prognosis, is characterized by high mortality because it is typically in an advanced stage at diagnosis and leaves very little hepatic functional reserve. Despite advances in medical and surgical techniques, there is no omnipotent tool that can diagnose HCC early and then cure it medically or surgically. Several recent studies have shown that a variety of pathways are involved in the development, growth, and even metastasis of HCC. Among a variety of cytokines or molecules, some investigators have suggested that arrest-defective 1 (ARD1), an acetyltransferase, plays a key role in the development of malignancies. Although ARD1 is thought to be centrally involved in the cell cycle, cell migration, apoptosis, differentiation, and proliferation, the role of ARD1 and its potential mechanistic involvement in HCC remain unclear. Here, we review the present literature on ARD1. First, we provide an overview of the essential structure, functions, and molecular mechanisms or pathways of ARD1 in HCC. Next, we discuss potential clinical implications and perspectives. We hope that, by providing new insights into ARD1, this review will help to guide the next steps in the development of markers for the early detection and prognosis of HCC.

## Liver cancer: The search for an early-stage indicator

A protein that is highly expressed in cancer with extensive blood vessel development may provide a potential biomarker for early-stage liver cancer. Liver cancer is often not diagnosed until it is advanced and is also hard to be cured despite of advances in treatment, meaning patients often die from the disease. No tools for early detection or prognosis prediction exist, and scientists are keen to find useful biomarker molecules. Young-Hwa Chung at the University of Ulsan College of Medicine, Asan Medical Center, Seoul, and co-workers in South Korea reviewed recent research into one possible cancer-related protein, arrest-defective 1 (ARD1), known to be highly expressed in certain cancers and possibly associated with poor prognosis. While ARD1 appears to regulate pathways critical to cancer progression and promote cancer cell invasiveness, further in-depth investigations are needed to clarify its specific role in liver cancer.

## Introduction

Arrest-defective 1 (ARD1) was originally identified as an N-terminal acetyltransferase (NAT) that catalyzes N-terminal acetylation of proteins and has lysine acetyltransferase (KAT) activity. Various isoforms of ARD1 have been identified in mammals according to differences in amino acid number, including ARD1^235^, mARD1^225^, ARD1^198^, and ARD1^131^. Each isoform has a different biological function^1,2^.

ARD1 is involved in various cellular functions that regulate cell division, proliferation, and tumorigenesis. Furthermore, ARD1 is involved in normal development and viability^3–6^. ARD1 also appears to have a role in brain development. In particular, ARD1 plays an important role in the development of neuronal dendrites and is likely involved in neurological diseases^7–9^. ARD1 is also implicated in either susceptibility to or protection against oxidative stress^9,10^.

Several reports have linked increased ARD1 expression to various human cancers, such as breast, prostate, lung, liver, cervical, bladder, and colorectal cancers^11–14^. Further, higher ARD1 expression is associated with poorer outcomes among cancer patients, including lower survival and more aggressive tumors^11,13^.

Hepatocellular carcinoma (HCC), which is common in Korea and representative of malignancies with a poor prognosis, is characterized by high mortality because it is typically in an advanced stage at diagnosis and because it leaves a poor hepatic functional reserve. To date, the potential role and significance of ARD1 in HCC carcinogenesis is unknown. In support of a potential link, one study showed higher ARD1 expression in HCC tissue than in non-HCC tissue, and patients with higher ARD1 expression also showed more frequent microvascular invasion (MVI) than patients with lower ARD1 expression^15^. We previously showed that ARD1 overexpression was associated with MVI, poor differentiation of tumors, a high cumulative recurrence rate, and poor survival^16^. Another report has suggested that ARD1 expression plays an important role in the development, progression, and potential recurrence of HCC^17^.

The present review provides a summary of recent reports regarding ARD1 and gives an overview of the essential structure, functions, and molecular mechanism or pathways of ARD1 in HCC carcinogenesis. We also discuss potential clinical implications and perspectives, such as the potential of ARD1 as a candidate tumor marker or a future target for cancer therapy.

## Basic concepts concerning ARD1

### Discovery of ARD1

ARD1 was first identified in *Saccharomyces cerevisiae* by Whiteway and Szostak in 1985^18^. As its full name of “arrest-defective 1” suggests, yeast ARD1 is considered to have an important role in the cell cycle and cell survival. Mutations of yeast ARD1 are associated with defects in entering the stationary phase and sporulating under nutrient-limited conditions, indicating that ARD1 is involved in controlling the switch between mitosis and alternative cell fates^18–20^.

ARD1 is highly conserved across organisms, from yeast to mammals. Mammalian ARD1 is located on chromosome Xq28 and regulates diverse cellular functions, including the cell cycle, cell migration, apoptosis, autophagy, differentiation, development, and diseases^7,21–25^. Therefore, ARD1 has garnered attention for its potential role in the development of cancer^26–28^. Indeed, ARD1 is upregulated in various human cancers such as lung, breast, prostate, and colorectal cancers, and its expression level is strongly associated with clinical outcomes among cancer patients^13,14,29–31^. Furthermore, several mechanistic studies have revealed that ARD1 directly or indirectly regulates diverse cellular pathways that are known critical steps in the progression of cancer^21,25,32–34^. Based on this evidence, ARD1 has emerged as a promising target for cancer therapy.

### ARD1 variants

In mammal, various isoforms of ARD1 have been identified, including ARD1^235^, ARD1^225^, ARD1^198^, and ARD1^131^ ^1,2^. ARD1 variants are given their names based on their different numbers of amino acids; the isoforms ARD1^235^, ARD1^225^, ARD1^198^, and ARD1^131^ contain 235, 225, 198, and 131 amino acids, respectively. Among these variants, ARD1^235^ and ARD1^225^ have been most extensively characterized in human and mouse cells.

**Fig. 1:**
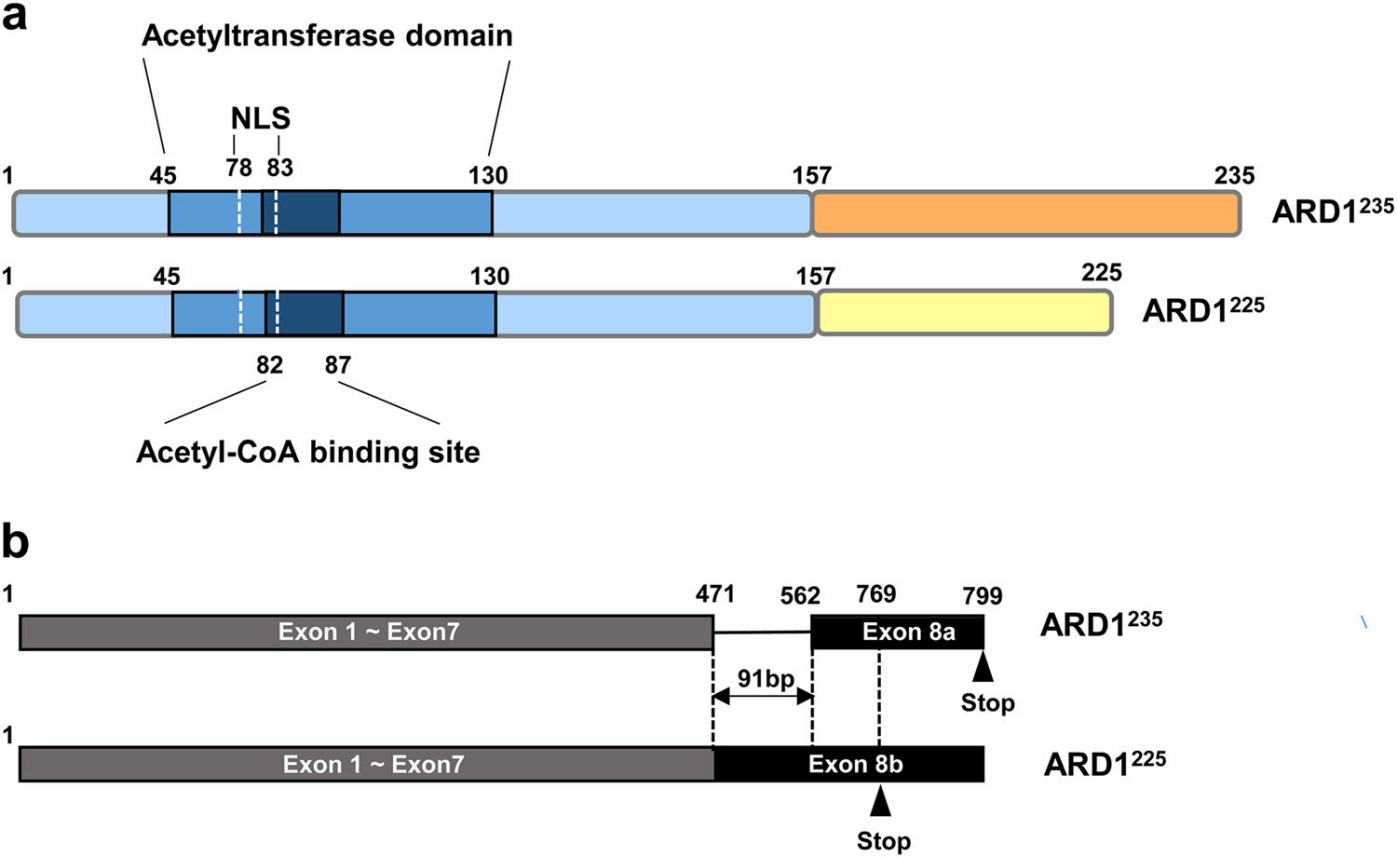
The structure of ARD1. **a** Structures of ARD1 isoforms. The ARD1^235^ and ARD1^225^ isoforms share a highly conserved N-acetyltransferase domain located between amino acids 45 and 130 (aa 45–130). The N-acetyltransferase domain contains an acetyl-CoA-binding site (RRLGLA) located at aa 82–87 and a nuclear localization signal (NLS) (KRSHRR) located at aa 78–83. ARD1^235^ and ARD1^225^ have completely different amino acid sequences in their C-terminal region, starting at aa 158. **b** Alternative splicing of ARD1 mRNA. ARD1 variants are derived from alternative RNA splicing. Alternative splicing at exon 8 alters the reading frame and introduces stop codons at amino acids 235 and 225, resulting in the production of two ARD1 isoforms, ARD1^235^ and ARD1^225^, respectively

ARD1^235^ and ARD1^225^ isoforms share a well-conserved N-acetyltransferase domain located between amino acids 45 and 130 (aa 45–130), although ARD1^131^ and ARD1^198^ have only partial N-acetyltransferase domains. The N-acetyltransferase domain is considered a core region of ARD1 because it contains an acetyl-CoA-binding site (RRLGLA) located at aa 82–87 that is critical for the acetyltransferase enzyme activity of ARD1. The N-acetyltransferase domain also contains a nuclear localization signal (NLS) (KRSHRR) located at aa 78–83, indicating a potential for dynamic translocation of ARD1 between the cytosol and the nucleus (Fig. 1a)^1,21^.

Whereas N-terminal domains are well-conserved in ARD1 variants, the variants have completely different amino acid sequences in C-terminal regions due to alternative RNA splicing. Alternative splicing at exon 8 alters the reading frame and introduces stop codons at amino acids 235 and 225, resulting in the production of two ARD1 isoforms, ARD1^235^ and ARD1^225^, respectively (Fig. 1b). Although nucleotide sequences of ARD1^225^ are identical to those of ARD1^235^ except nucleotides 472–562 of exon 8, ARD1^235^, and ARD1^225^ have different amino acid sequence in their C-terminal region, starting at amino acid 158 (aa 158).

Interestingly, mouse and human cells express different types of ARD1 variants. While human cells express two ARD1 variants (i.e., ARD1^235^ and ARD^131^), mouse cells express three (ARD1^235^, ARD1^225^, and ARD1^198^)^1^. Thus, ARD1^225^ is not expressed in humans, and mouse cells express both ARD1^225^ and ARD1^235^. In contrast to mouse genomic DNA, human genomic DNA from ARD1 has no acceptor site for alternative splicing of exon 8. Thus, the ARD1^225^ transcript cannot be produced from the human genome. These data suggest that the regulation of alternative splicing of ARD1 RNA might be evolved differently between the species^2^.

Given the variation in amino acid sequence within C-terminal domains, it is not surprising that different ARD1 variants are associated with different functional characteristics. Indeed, while ARD1^225^ inhibits angiogenesis under hypoxic conditions, ARD1^235^ has little relationship with tumor angiogenesis^1,35^. Instead, ARD1^235^ promotes cell growth under normoxia, whereas  ARD1^225^ has no effect on cell growth^35^. The different biological functions of ARD1 variants might reflect their different subcellular compartments. In mouse cells, ARD1^225^ and ARD1^235^ proteins are present in the cytoplasm and nucleus, respectively^2^. In human cells, several groups have reported the subcellular localization of ARD1^235^; however, those results have been ambiguous and discrepant. Earlier studies reported that the majority of human ARD1^235^ expression is in the nucleus, with low levels of expression in the cytoplasm^7,21^. By contrast, other studies suggest that human ARD1^235^ is predominantly located in the cytoplasm rather than in the nucleus^13,36^. In light of these discrepancies, the subcellular localization of human ARD1^235^ appears to differ based on the cell line, suggesting that localization of human ARD1^235^ is dependent on the cellular context^28^. Recently, Park et al. provided one possible explanation for these complicated and often conflicting results^37^. They found that human ARD1^235^ is imported to the nucleus of growing cells, especially during the S phase of the cell cycle. Furthermore, nuclear translocation of ARD1^235^ is mediated by NLS, and this translocation is essential for proper cell cycle progression. Based on these results combined, ARD1 appears to have variable properties depending on subcellular localization, specific isoform, and physiological conditions. Therefore, it will be critical to consider the role of these factors in modulating ARD1 activity when evaluating the potential for cancer treatment that targets ARD1.

### ARD1 as an acetyltransferase

#### N-terminal acetyltransferase activity

ARD1 was originally identified as a NAT that catalyzes N-terminal acetylation of proteins^18^. NATs transfer acetyl groups from acetyl-CoA to the first amino acid of a given protein. N-terminal acetylation is one of the major protein modifications in eukaryotes, occurring in 80–90% of proteins in humans and in 50–70% of proteins in yeast^19,38^. When proteins are synthesized and extruded from the ribosome, NATs associated with the ribosome acetylate the N-terminal α-amino group of the nascent polypeptide^39^. Thus, protein N-terminal acetylation occurs co-translationally and irreversibly.

To date, six human NAT complexes have been identified: NatA, NatB, NatC, NatD, NatE, and NatF. The naming of each complex reflects the specific amino acid sequences contained in the N-terminus. ARD1 is a catalytic subunit of the NatA complex that acetylates Ser, Ala, Gly, Thr, and Val within the N-terminus of a protein after an initiator methionine is cleaved by aminopeptidase^27^. N-terminal acetylation is thought to play a role in protein stability by inhibiting protein degradation via blockade of N-terminal ubiquitination^40^. Indeed, ARD1 acetylates the first methionine of the tuberous sclerosis 2 (TSC2) protein, which, in turn, stabilizes the TSC2 protein^28^. However, other studies have reported that N-terminal acetylation does not block protein degradation. In fact, N- terminal acetylation of a protein may even create specific degradation signals^41^. Beyond protein stability, several studies report other potential roles of protein N-terminal acetylation, including protein–protein interactions and targeting subcellular localization^42,43^. Taken together, the existing data imply that the role of N-terminal acetylation may differ based on the substrate protein.

Recently, N-terminal acetylation activity by ARD1^235^ was found to relate strongly to genetic disease in humans. In particular, a Ser37Pro mutation of ARD1^235^ was recently discovered in Ogden syndrome, resulting in the lethality of male infants^44^. Subsequent in vitro experiments have revealed that the Ser37Pro mutation significantly reduces the N-terminal acetylation activity of ARD1^235^, suggesting a potential link between the NAT function of ARD1^235^ and human disease^44–46^.

#### Lysine acetyltransferase activity

While co-translational N-terminal acetylation is one of the most common protein modifications, a less common but potentially more important form of protein acetylation occurs post-translationally on lysine residues via KATs. The addition of an acetyl group to lysine prevents the formation of positive charges on the amino group. This modification therefore has a significant impact on the electrostatic properties of the protein. Lysine acetylation is crucial for regulating various protein functions, including chromatin-based transcriptional control, protein–protein interactions, and protein stability^47^.

Although ARD1 was originally identified as a NAT, its subcellular localization in non-ribosomal forms and in the nucleus strongly suggests that ARD1 has other functions. Indeed, mammalian ARD1 has both NAT and KAT activity. Mouse ARD1^225^ was first proposed to possess KAT activity because it acetylated the lysine 532 residue of HIF-1α, resulting in the degradation of HIF-1α via the ubiquitin–proteasome system^48^. Thereafter, human ARD1^235^ was also shown to have KAT activity. Human ARD1^235^ acetylates β-catenin and is involved in the proliferation of lung cancer cells, although the target site of lysine acetylation has not yet been identified^49,50^. ARD1 post-translationally mediates the acetylation of a microtubule fraction purified from porcine brain in vitro^8^. However, the specific substrate from the microtubule fraction containing α/β-tubulin has not been identified. Furthermore, ARD1^235^ acetylates myosin light chain kinase (MLCK), deactivating that enzyme^51^. In prostate cancer cells, ARD1 acetylates the androgen receptor (AR) and this acetylation is required for AR-mediated gene transcription^30^. Recent studies have uncovered substrate proteins of ARD1 and their specific target sites of lysine acetylation. In particular, during bone formation, ARD1 acetylates the lysine-225 residue of Runt-related transcription factor 2 (Runx2). Runx2 acetylation, in turn, leads to the inhibition of Runx2-mediated gene transcription, which results in the regulation of osteoblast differentiation^52^. Recently, Heat Shock Protein 70 (Hsp70) was identified as a new substrate of ARD1. Under stress conditions, the lysine 77 residue of Hsp70 is acetylated by ARD1, and this acetylation is essential for the enzyme activity of Hsp70 and control stress response of cells^9^. Furthermore, under glutamine deprivation, ARD1 acetylates the lysine-388 residue of phosphoglycerate kinase 1 (PGK1), a metabolic enzyme. PGK1 acetylation via ARD1 enhances kinase functions of PGK1 and contributes to the maintenance of cellular homeostasis in a nutrient-starved state^53^. Based on evidence regarding the KAT activity of ARD1, it is notable that ARD1 is the first identified protein with both NAT and KAT activity. However, whether KAT and NAT activity of ARD1 are regulated in similar or different ways remains unclear.

### Biological function of ARD1

#### ARD1 in non-cancerous conditions

ARD1 is involved in various cellular functions (Table 1). In particular, emerging research has shed new light on the importance of ARD1 during developmental stage. Depletion or mutation of ARD1 leads to lethality in *Drosophila*, *Danio rerio*, *Caenorhabditis elegans*, and *Trypanosoma brucei*, highlighting an essential role for ARD1 in typical development and viability^3–6^. Dysfunction of ARD1 is also fatal in humans, given that ARD1 mutations that cause a reduction in NAT activity can result in infant mortality, intellectual disability, development delay, and Lenz microphthalmia syndrome^44,54–56^. In mouse models, mice with depleted levels of the ARD1^225^ and ARD1^235^ variants display severe defects in bone development^52^. ARD1^235^ is also implicated in brain development. Mouse models suggest that ARD1^235^ is expressed in regions of cell division and migration throughout the course of brain development, and expressions of these proteins is downregulated as neurons mature and form appropriate synaptic connections^57^. Similarly, downregulation of ARD1^235^ is observed during differentiation of neurons, suggesting that ARD1^235^ plays an important role in development of dendrites in the brain^8^.

**Table 1:** The biological functions of ARD1

Category	Function	Reference
Development	Viability of *Trypanosoma brucei*	Ingram et al.^4^
	Survival and proliferation	Wang et al.^6^
	Survival and metabolism of C elegans	Chen et al.^3^
	Normal development and viability of zebrafish	Ree et al.^5^
	Ogden syndrome in males	Rope et al.^44^
	Intellectual disability	Rauch et al.^56^
	Lenz microphthalmia syndrome	Esmailpour et al.^54^
	Global developmental delay	Popp et al.^55^
	Bone development	Yoon et al.^52^
	Brain development	Sugiura et al.^57^
	Neuronal dendritic development	Ohkawa et al.^8^
Neuronal disorders	Relation to neuronal development	Asamui et al.^7^
	Protection of dopaminergic neurons in zebrafish	Seo et al.^9^
Oxidative stress	Promoting oxidative injury by MSRA acetylation	Shin et al.^10^
	Maintaining protein homeostasis by Hsp70 acetylation	Seo et al.^9^

In addition to development, ARD1 is implicated in various neurological diseases. In particular, through direct interaction with amyloid-β protein (Aβ), ARD1^235^ suppresses the secretion of Aβ, which is the first step in the development of Alzheimer’s disease^7^. Another study using a zebrafish model of Parkinson’s disease showed that ectopic expression of dominant-negative human ARD1^235^ exacerbated dopaminergic neuronal loss and locomotor deficits compared with wild-type human ARD1^235^ expression^9^. These results suggest that ARD1^235^ may be protective against the development of neurodegenerative disease.

Recent studies have revealed that ARD1 is also involved in cellular response to oxidative stress. ARD1^235^ represses the enzymatic activity of methionine sulfoxide reductase A, an enzyme that protects cells from oxidative stress by removing reactive oxygen species. In support of a pro-oxidant role of ARD1^235^, an ARD1^235^ transgenic mouse is more susceptible to oxidative stress and shows more severe tissue injury compared with wild-type mouse^10^. However, results of a recent study suggest the opposite effect-that ARD1^235^ may be protective against oxidative stress. In this study, ARD1^235^ prevented protein aggregation under oxidative stress by regulating chaperone function of Hsp70 required for the maintenance of protein homeostasis. The authors therefore concluded that ARD1^235^ protects cells against oxidative stress and functions as an anti-oxidant protein^9^. Although it is presently unclear whether ARD1 has a protective or apoptotic role under oxidative stress, existing data suggest that ARD1 is a potential target for ameliorating oxidative stress in diverse human health conditions, such as cancer, neurological disease, inflammation, and metabolic disease.

#### ARD1 in cancerous conditions

##### Role of ARD1 as a tumor suppressor

Mammalian ARD1 was first reported to act as a tumor suppressor, given that mouse ARD1^225^ inhibits HIF-1α stabilization and angiogenesis by acetylating the lysine-532 residue^48^. In contrast to ARD1^225^, ARD1^235^ has no effect on HIF-1α acetylation or stability. However, a crucial role of ARD1^225^ in HIF-1α stability was confirmed using a mouse tumor model^11^. In that study, the authors generated Apc^Min/+^/ARD1^225^ transgenic mice and demonstrated the anti-tumor effect of ARD1^225^ in mice. In particular, they found a significant decrease in the quantity of intestinal polyps among Apc^Min/+^/ARD1^225^ transgenic mice compared with Apc^Min/+^ transgenic mice. Within the intestinal polyps of Apc^Min/+^/ARD1^225^ transgenic mice, they found that VEGF expression was also reduced, which, in turn, suppressed tumor angiogenesis and growth.

In addition to ARD1^225^, human ARD1^235^ is also reported to have tumor suppressor properties. In breast cancer, higher ARD1^235^ expression levels are correlated with better clinical outcome from the patients patients, including higher survival, smaller tumor volume, and lower rates of lymph node metastasis^34,58^. In lung and gastric cancers, ARD1^235^ expression is significantly downregulated in malignant tumors with lymph node metastasis compared with primary tumors without metastasis, suggesting that downregulation of ARD1 plays a causal role in tumor development^29^.

Investigations into the underlying molecular mechanisms have led to new insights into mechanisms through which ARD1 reduces proliferation and metastasis. In breast cancer cells, inhibition of ARD1^235^ induces autophagy by inhibiting mTOR signaling and subsequently reduces cancer cell growth^58^. Yi et al. reported that ARD1^235^ is required for caspase activation during apoptosis and that depletion of ARD1^235^ may thus provide robust protection against DNA damage-induced cell death^25,59^. These data suggest a pro-apoptotic role of ARD1^235^. Regarding cell motility, ARD1^235^ directly acetylates the lysine 608 residue of MLCK, leading to the inhibition of MLCK activity required for cell contraction and migration^51^. In lung cancer cells, ARD1^235^ binds to p21-activated kinase-interacting exchange factor to block its downstream Rac1/Cdc42 pathway. Blockade of the Rac1/Cdc42 pathway results in the inhibition of cancer cell metastasis, although this effect appears to be independent of the acetyltransferase activity of ARD1^235^ ^29^. In breast cancer cells, ARD1^235^ inhibits cancer cell migration by targeting signal transducer and activator of transcription 5a (STAT5a) independently of acetyltransferase activity-independent manner^34^. Taken together, although ARD1 is an acetyltransferase enzyme, these results suggest that ARD1^235^ can exert oncogenic functions, independent of its catalytic activity.

##### Role of ARD1 as an oncogene

**Fig. 2:**
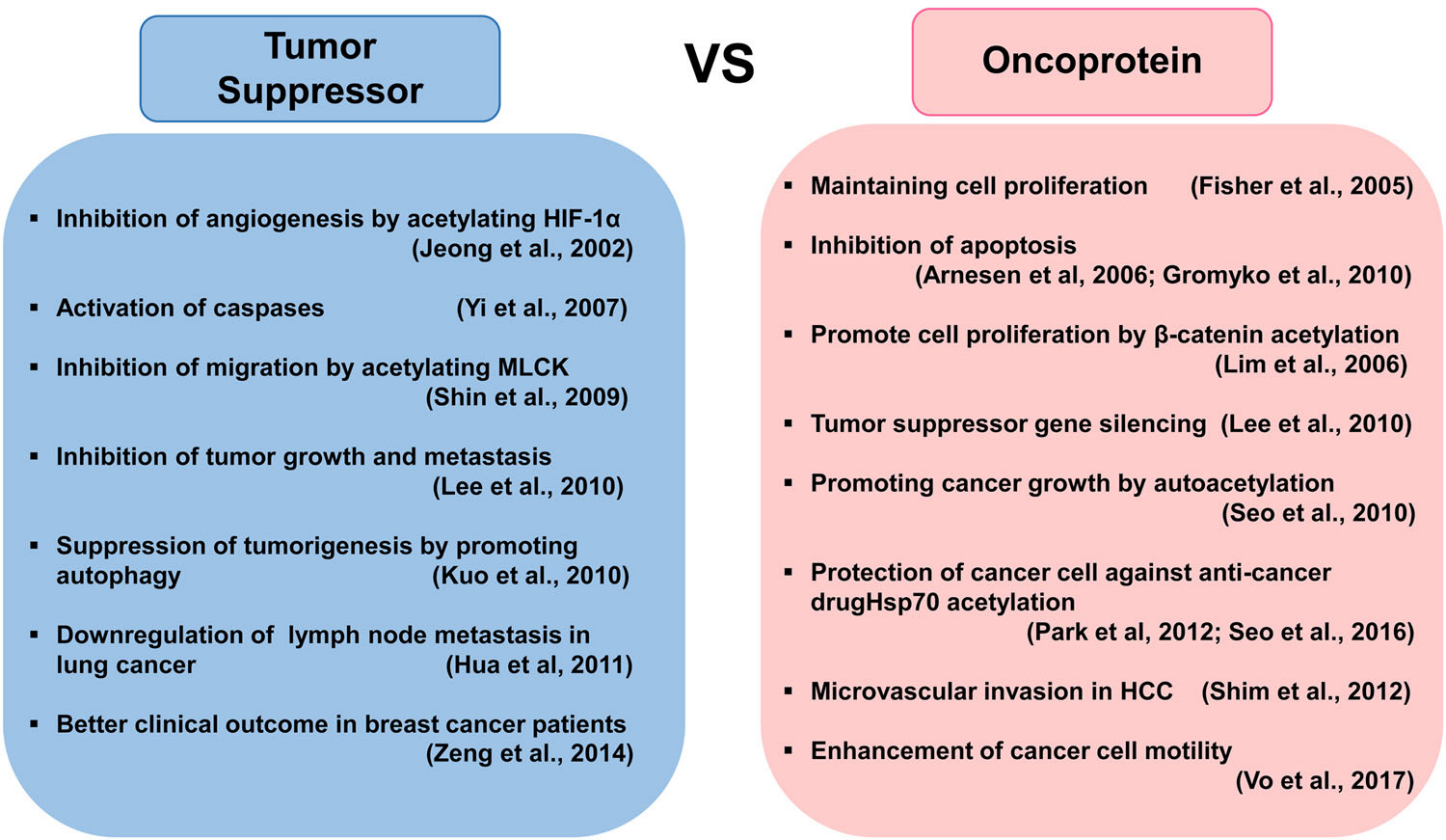
Controversial roles of ARD1 in cancer development and progression. Many studies have reported an important role of ARD1 in cancer progression. However, it is still debated whether human ARD1 serves as an oncogene or a tumor suppressor

Although several studies have demonstrated that ARD1 is an important molecule involved in cancer progression, it is still under debate whether human ARD1^235^ serves as an oncogene or a tumor suppressor. In fact, a growing number of studies have highlighted potential oncogenic properties of ARD1 in cancer progression (Fig. 2). At present, it is widely accepted that ARD1^235^ relates positively to cellular growth. Fisher et al. first suggested that ARD1^235^ is required to maintain division and proliferation of liver cancer cells under hypoxia^60^. Furthermore, a fluorescence-activated cell sorting analysis revealed that ARD1^235^ promotes cell proliferation by regulating the G1/S and G2/M checkpoints of the cell cycle in breast and lung cancer cells, indicating that ARD1^235^ is relevant for cell proliferation^14,32,50^. Regarding the molecular mechanisms, ARD1^235^ promotes tumor growth by enhancing cyclin D1 expression, and these effects are mediated by the activation of β-catenin and activator protein 1^32,50^. Recently, SAM domain and HD domain-containing protein 1—the latter of which is involved in the regulation of intracellular dNTP pools required for DNA replication—were found to be involved in ARD1^235^-mediated cancer cell growth^61^.

Indeed, a number of links have been found between increased expression of ARD1^235^ and human cancers, such as breast, prostate, lung, liver, cervical, bladder, and colorectal cancers^12–14,62^. In addition, high levels of ARD1^235^ expression correspond with poorer outcomes among cancer patients, such as low survival rate and more aggressive tumors^13,62^. These point to the potential role of ARD1^235^ as an oncoprotein.

In addition, ARD1^235^ seems to promote the invasive properties of cancer cells. In lung cancer cells, ARD1 downregulates E-cadherin by regulation of DNA methyltransferase 1^62^. In breast cancer cells, ARD1^235^ enhances cell motility by activation of Aurora A kinase function^33^. Based on these results, ARD1^235^ expression is closely related to MVI in HCC patients^15^.

Other studies demonstrate that ARD1^235^ has a protective effect against cell death. Depletion of ARD1^235^ induces the transcription of p53-dependent pro-apoptotic genes and activation of the caspase-dependent apoptosis pathway^22,63^. Depletion of ARD1^235^ also revokes doxorubicin—but not tumor necrosis factor α (TNFα)-induced activation of the nuclear transcription factor-κB (NF-κB)^64^. Neuroblastoma cells expressing dominant-negative ARD1^235^ are more sensitive to etoposide compared with wild-type ARD1-expressing cells^9^. These results suggest that ARD1^235^ is involved in drug-resistant mechanisms of cancer cells. Thus, ARD1^235^ could be a potential marker for the prediction of chemoresistance in cancer patients. In-depth studies are needed to address the association between ARD1^235^ expression and drug sensitivity or resistance of cancer patients and to elucidate the detailed molecular mechanism by which ARD1^235^ protects cancer cells against anti-tumor drugs.

## General consideration of ARD1 in clinical respects

HCC is the malignancy with the poorest prognosis among the most common types of cancers^65^. In line with this poor prognosis, the majority of HCC patients are hardly candidates for the best curative option, curative surgical resection, because of frequent vascular invasion and/or intrahepatic or extrahepatic metastases at the time of diagnosis^66^. Poorly reserved underlying liver function and frequent postoperative recurrence of HCC represent additional hindrances to successful disease management^67^. To expand the understanding of potential clinical applications of ARD1, now we are going to enter into the clinical implications of ARD1, which will be focused in a field of HCC.

### Basic clinical concepts of ARD1 in hepatocarcinogenesis

HCC is typically characterized by hypervascularity. Hypervascularity may be due to the fact that HCC typically develops in hypoxic environments caused by underlying chronic liver diseases, such as liver cirrhosis. In most HCCs, underlying chronic liver diseases necessarily provoke an alteration (i.e., decrement) in intrahepatic blood flow, which results in poor oxygen supply (i.e., hypoxia) to the liver. This hypoxia accelerates the production of angiogenic factors, including VEGF, basic fibroblast growth factor, and insulin-like growth factor-II (IGF-II). Overexpression of angiogenic factors subsequently promotes neovascularization, which further contributes to the development of HCC and leads to increased vascular density in tumors (i.e., hypervascular tumors). At the same time, overexpression or hyperactivation of angiogenic factors can enhance the growth of HCC and also promote intrahepatic or extrahepatic metastases^68^.

In previous studies, we sought to identify markers for the early detection and prediction of post-treatment prognosis among HCC patients using serological or histological features. Although we demonstrated their clinical significance and found their usefulness in clinical practice, the many ways to the destination mean that there is no single, perfectly effective way to cover all of them. Thus, it is essentially impossible to understand all forms of hepatocarcinogenesis and to cure HCC completely by conquering a sole pathway because it is too complex^68,69^. Indeed, we found that several factors are involved in hepatocarcinogenesis, including IGF-II, TNF-α, transforming growth factor beta 1 (TGF-β), osteopontin, metastatic tumor antigen (MTA) 1, and MTA2. These markers can be used not only for diagnosis/prognosis but also as therapeutic targets for postoperative adjuvant therapies^70–80^.

A separate line of work suggests that hypoxia is associated with tumorigenesis. In particular, HIF-1α has been reported to play a critical role in hepatocarcinogenesis^81^. In HCC, intratumoral HIF-1α and VEGF expression significantly increase to promote new vessel formation by stimulating transcription of cytokines, which are involved in angiogenesis^82^. Relying on various conditions, HIF-1α reacts differently according to the oxygen concentration in the tissues^83^. Under normoxia, HIF-1α typically has a short half-life of 5 min; thus, it is immediately degraded into biologically inactive products. That is, under normoxia, the proline residues at P402 and P564 of the ODD domain of HIF-1α undergo hydroxylation, then ubiquitination by the von Hippel-Lindau (pVHL) protein, followed by degradation via a proteasome. At this step, the ODD domain of HIF-1α plays an important role in degradation as well as the prevention of its overexpression^84–86^. In contrast to normoxia, hypoxia stabilizes HIF-1α to accelerate the development, growth, and subsequent metastasis of cancers via stimulating the transcription of angiogenic factors (e.g., VEGF)^87^. These results provide a theoretical basis for notion that the HIF-1α pathway is a promising target for cancer therapies^88^.

As a first step, it may be worth briefly mentioning the association between HIF-1α and MTA1 in hepatocarcinogenesis. For more in-depth discussion, we refer the reader to previous papers^80,89,90^. Based on our prior studies, we found that MTA1 is more highly overexpressed in hepatitis B virus (HBV)-associated HCC than in non-HBV-associated HCC^76^. This may be due to the HBx protein of HBV that can induce MTA1 expression and result in the enhancement of hypoxia signaling via HIF-1α stabilization. Consequently, stabilization of HIF-1α accelerates angiogenesis under hypoxia by reducing the binding of pVHL protein to HIF-1α and preventing the ubiquitin-dependent degradation of HIF-1α. Furthermore, MTA1 itself may increase the transcription and stabilization of HIF-1α^80^. MTA1 activation by TGF-β1 is also involved in tumor progression through the promotion of epithelial–mesenchymal transition (EMT)^91,92^.

On the other hand, it seems that the association between HIF-1α and ARD1 in human HCC is not yet clear. In the acetylation process, ARD1 which exists in all mammalian cells including brain, heart, bone marrow, and liver, acts as a key molecule. ARD1 was named because its deletion impairs cell mitosis and arrests cell division. In particular, ARD1 is detected in high concentrations in tumor cells of colorectal and breast cancers in some patients^1,21^. Furthermore, concentrations of ARD1 are high in organs that are highly vascular (e.g., heart, liver), and thus, ARD1 may play an important role in determining the destiny of cells during cell cycles. It, therefore, is thought of ARD1 that it should be deeply involved in the progression of malignancies as the cell division increases.

The existing data, in vitro, suggest that mouse ARD1 plays a pivotal role as a negative controller of HIF-1α stability under normoxia^48,93^. It is assumed that mouse ARD1 acetylates HIF-1α protein by direct reaction with Lys_532_ of the ODD domain. This deepens the instability of HIF-1α and then suppresses the expression of angiogenic factors such as VEGF, which lead to be able to prevent development and/or progression of malignancies by inhibiting neovascularization. On the other hand, recent reports suggest that human ARD1 may have no impact on the stability of HIF-1α^36,94^. In addition, there is a view that the ARD1 is not regulated by hypoxia in several human tumor cell lines including cervical adenocarcinoma, fibrosarcoma, and breast cancer cells.

Notably, as mentioned before, HCC is a typical hypervascular tumor and also it has been developed generally in hypoxic condition such as liver cirrhosis. Additionally, the HIF-1α signaling pathway is the representative crucial mechanism in hepatocarcinogenesis and angiogenesis. Although, to date, the biologic functions of ARD1 have not been clearly defined in malignancies, especially HCC, and the direct association of ARD1 with HIF-1α in HCC of human is not also clarified, we still wonder whether ARD1 will have a direct or indirect relationship with HIF-1α in human hepatocarcinogenesis.

On the basis of the facts described above, it is possible that ARD1 is also implicated in the development of HCC and its progression, including tumor growth and metastasis. Therefore, it is very valuable to investigate ARD1 expression and to ascertain its potential association with clinical characteristics for clarifying hepatocarcinogenesis and setting up the preventive strategies in the future. At the same time, it will be useful to amend current treatment modalities and develop new effective therapies.

### ARD1 expression and its clinical significance in non-HCC neoplasia

Dose ARD1 act as an oncoprotein or a tumor suppressor in human cancers? This point remains to be clarified, but a number of in vitro studies on the molecular mechanisms of carcinogenesis have been published in favor of the oncoprotein role^27,80,89,90^. Research on ARD1 in clinical settings, however, remains in its very early stages. In fact, there are very few reports on ARD1 using human neoplastic tissues. An even smaller number of studies examined the potential association between ARD1 expression and recurrence or survival in patients with cancers^13–17,95–98^. These studies are summarized in Table 2.

**Table 2:** Clinical studies of ARD1 overexpression in malignancies including hepatocellular carcinoma in humans

	Subjects	Methods of evaluation	Key findings
Yu et al.^14^	19 common types of cancers (*n* = 400)^a^	Immunohistochemistry	ARD1 had a significantly positive correlation with common cancers overall, including individual breast and colorectal cancers
Ren et al.^13^	Colorectal cancer (*n* = 50)	Immunohistochemistry	82% (41/50) were ARD1 positive
Jiang et al.^96^	Colorectal cancer (*n* = 270)	ELISA using anti-ARD1A antibody (serum) and immunohistochemistry (tissue)	Anti-ARD1A antibody can be detected in serum from patients with colon cancer. ARD1A is a novel tumor-associated antigen and a prognostic factor for colon cancer
Liu et al.^97^	Colon adenocarcinoma (*n* = 225)	Immunohistochemistry	When combined with other tumor markers, ARD1 increased the overall detection accuracy and prognostic value
Wang et al.^98^	Breast cancer (*n* = 356: 82 malignant vs. 274 benign)	Immunohistochemistry	ARD1 overexpression is correlated with breast cancer and especially with lymph node metastasis and estrogen receptor positivity
Huang et al.^95^	HCC (*n* = 112)	Microarray for single-nucleotide polymorphisms (SNPs)	Loss of heterozygosity in ARD1B was associated with significantly decreased survival
Shim et al.^15^	HCC (*n* = 94)	Real-time PCR (mRNA)	Intratumoral ARD1 mRNA levels were associated with microvascular invasion but not with 5-year recurrence-free or overall survival
Lee et al.^16^	HCC (*n* = 698)	Immunohistochemistry	ARD1 overexpression was closely associated with frequent postoperative recurrence and poor survival
Kang et al.^17^	DN (*n* = 51), HCC (*n* = 226)	Immunohistochemistry	ARD1 expression reflects the malignant potential of dysplastic hepatic nodules

^a^Total sample (*N* = 400; *p* < 0.005); urinary bladder, *n* = 21; breast, *n* = 48 (*p* *<* 0.05); cervix, *n* = 14; hypothyroid, *n* = 12; stomach, *n* = 56; lung, *n* = 29; colorectum, *n* = 125 (*p* *<* 0.01); prostate, *n* = 12; nasopharynx, *n* = 11 (*p* *<* 0.1); liver, *n* = 9; esophagus, *n* = 19; skin, *n* = 14; kidney, *n* = 10 (*p* *<* 0.1); gallbladder, *n* = 4; eye, *n* = 3; vocal cords, *n* = 5 (*p* *<* 0.1); pancreas, *n* = 2; ovary, *n* = 3; brain, *n* = 3

Yu et al. investigated human ARD1 expression in cancer tissues and compared this expression to that in non-neoplastic tissues^14^. For this study, the investigators enrolled a relatively large number of total subjects (400 with common cancers vs. 133 with matching non-cancerous tissues). Across the sample, the rates of ARD1-positive tissue were 52.3% (209/400) of cancer tissues and 31.5% (42 of 133) of non-cancerous tissues. Although the overall difference in ARD1-positive rates between cancer and non-cancerous tissues reached statistical significance (*p* *<* 0.005), ARD1 was still detected in a substantial number of non-cancerous tissues (i.e., 31.5%), and this rate was not significantly different from specific cancer types (refers to footnote of Table 2). This is because only small number of cancer cases of each type were merged into the same overall group and then analyzed as a whole. Across the cancer types, there was a significant association between ARD1 overexpression and colorectal (*n* = 125, 48.0% vs. 18.2%; *p* *<* 0.01) and breast cancers (*n* = 48, 62.5% vs. 38.1%; *p* *<* 0.05). At any rate, the study confirmed that ARD1 expression is widespread in various cancers and, at the very least, showed its close association with colorectal and breast cancers. A link between ARD1 expression and colorectal cancers is consistent with a previous study by Ren et al^13^. In that study, the authors used immunohistochemistry and compared ARD1 expression in colorectal cancer and colitis tissues. They found that ARD1 was positive in 41 out of 50 (82%) tested colorectal cancer tissues, but weakly positive in only 12 of 50 (24%) matched normal tissues (*p* *<* 0.001) and negative in all of 20 colitis tissues (*p* < 0.001). Importantly, they showed the possibility of ARD1 as a potential biomarker in patients with colorectal cancer or even other malignancies. In fact, in the next study by the same investigators, they acquired the result with ARD1 overexpression in 84.1% (227 out of 270) of tested colon cancer tissues and, strikingly, successfully isolated the autoantibody against ARD1 in serum obtained from patients^96^. They suggested for the first time that ARD1 is a novel tumor antigen in colon cancers and also has a potential to predict prognosis in the patients with colon cancer. In a subsequent study, the investigators used four autoantibodies (synuclein-gamma (SNCG), a human homolog of piwi (Hiwi), phosphatase of regenerating liver-3 (PRL-3), and ARD1) from 225 colon cancer patients, which were all isolated from serum, to evaluate whether the combination of them improve the values as tumor markers in terms of early detection and prognostic prediction of cancer^97^. Results showed that the combination of autoantibodies was associated with an elevated positive tumor marker rate (76.9% or 173/225) and an increased detection accuracy of cancer, with 61.9% (252/407) for SNCG and 82.6% (336/407) for the combination, with a hazard ratio 3.2 (*p* *<* 0.001) for poor prognosis. It is somewhat conclusive that ARD1 is clearly overexpressed and can be considered a tumor-specific antigen in colon cancer.

In terms of other malignancies, fewer studies have been reported, for which reason it is difficult to conclude whether ARD1 has a potential clinical role. Consistent in part with the present conditions, ARD1 is overexpressed in malignant breast neoplasia compared with benign conditions, e.g., fibroadenomas, hyperplasia, inflammatory breast diseases, and cysts. According to a study by Yu et al., the ARD1-positive rate was significantly higher in breast cancer tissues than in non-cancerous ones (62.5% (30/48) *vs*. 38.1% (8/21); *p* *<* 0.05)^14^. Their findings are comparable to the ARD1-positive rate reported by Wang et al. in invasive ductal carcinomas (61.0% or 50/82) compared with benign conditions listed above (48.8% or 119/244)^98^. Although they demonstrated the implications of ARD1 in malignant breast neoplasia, there were some limitations. Above all, the staining intensity was not graded, and the tissues of substantial proportion of benign diseases also stained positive for ARD1, which did not make ARD1 a suitable indicator for differentiating breast cancers from non-neoplastic diseases, i.e., a tumor-specific marker. Further, there was a report in contrast to it that ARD1 is a tumor suppressor in breast cancer by reducing cell proliferation and autophagy via inhibiting mTOR signaling pathway^58^. In thyroid cancer, as well, it remains inconclusive whether ARD1 is an oncogenic protein or a tumor suppressor^21,99^.

### ARD1 expression and its clinical significance in HCC

So far, to our knowledge, there has been only one clinical study showing ARD1 overexpression by immunohistochemistry in human HCC samples^14^. However, the subjects with HCC were composed of only a very small portion of the total study population (<3%, 9/400); therefore, positive rates of ARD1 detection could not be statistically compared between the HCC and non-HCC groups [33.3% (3/9) vs. 0% (0/1), *p* *=* not applicable]. Also, the analyses for clinical outcomes could not be done in HCC because there were no clinical data about the patients such as HCC stages or the patients’ survival. Although another microarray study showed the association of a specific single-nucleotide polymorphism (loss of heterozygosity in ARD1B) with clinicopathological characteristics and survival in HCC patients, it could not directly demonstrate the significance of ARD1 at the protein levels for the development, progression, and/or prognosis of HCC.

Recently, in light of the potential of ARD1 as a predictor of clinical outcomes, we published an important paper using 94 HCC patients^15^. In the study, we hypothesized that ARD1 is overexpressed in HCC but not in non-HCC and thus may predict patients’ clinical outcomes in terms of recurrence and survival. The theoretical basis has been sufficiently described above: the main feature of HCC is hypoxia in the central lesion, resulting in hypervascularity, which is mediated by HIF1-α pathway. In the study, we quantified ARD1 mRNA levels in HCC and matching non-HCC tissues using real-time PCR. Based on these results, patients were regrouped into high (*n* = 38) and low levels (*n* = 56) of ARD1 expression. We found that the high-expression group had more frequent MVI than the low-expression group (32% vs. 14%; *p* < 0.05). Regrettably, however, we could not see the findings that we expected in 5-year recurrence-free (34% vs. 46%, *p* *=* 0.98) and overall survival rates (76% vs. 73%, *p* *=* 0.52). Importantly, intratumoral ARD1 mRNA levels were significantly high in HCCs with MVI, suggesting novel potential links between ARD1 overexpression and the clinical courses of HCC patients.

Very importantly, in April 2017, we presented novel findings from two large-scale clinical studies on the significance of ARD1 in HCC at the 52nd Annual Meeting of the European Association for the Study of the Liver in Amsterdam, the Netherlands. In the first study, results clearly demonstrated ARD1 overexpression in HCC, and a good correlationship between ARD1 expression and postoperative recurrence and patient survival. We enrolled 698 histologically confirmed HCC patients (162 with liver transplantation and 536 with curative surgical resection). By grading the intensity of ARD1 immunohistochemical staining, we investigated the association of ARD1 with the histological and radiological characters of the tumors, as well as patient clinical outcomes. The results demonstrated that ARD1 was stainable in 87% (605/698) of tumors, and, as expected, ARD1 overexpression was positively associated with MVI (*p* *<* 0.001) and poor differentiation of the tumors (*p* *<* 0.001). Interestingly, the 5-year cumulative recurrence rates of HCC increased as the intensity of ARD1 expression in HCC tissues increased (*p* *<* 0.05). Further, the survival of the patients also decreased with higher ARD1 expression (*p* *<* 0.01)^16^. In another presentation, we showed that ARD1 expression may reflect the malignant potential of dysplastic nodules, a considerable portion of which subsequently leads to HCC^17^. In brief, 63% (32/51) were stained positive for ARD1 in dysplastic nodules, whereas 93% (210/226) of HCC were stained positive ARD1 and all normal livers tested were negative ARD1 (0/4). Importantly, in the subgroups of patients histologically defined as having low-grade or high-grade dysplasia, both ARD1-positive rate and its staining intensity were significantly higher in high-grade dysplastic nodules than in low-grade ones. Altogether, we found that higher intensity of ARD1 staining via immunohistochemistry is associated with higher malignant potential, even in the dysplastic stages. Taken together, the evidence suggests that ARD1 in the liver may play an important role both in the early stages of HCC development and in its progression to the advanced stages.

## Clinical prospective of ARD1 in HCC

It is established that ARD1 widely exists in mammalian cells and acts as an acetyltransferase by catalyzing protein acetylation, which serves to regulate cell division, proliferation, and even tumorigenesis^11^. All reviewed data are in accordance with clinical observations from all colorectal cancers and selected groups with breast neoplasia as summarized in Table 2. First, what we wish to emphasize here is that ARD1 overexpression in the liver continuously changes in a stepwise pattern from low-grade dysplasia, to high-grade dysplasia to HCC, as determined histologically. Moreover, intensity of ARD1 expression is well correlated with HCC recurrence and patient long-term survival. This means that ARD1 not only takes part in early development (i.e., from dysplasia to cancer) but also plays a key role in tumor progression (i.e., poor differentiation and MVI) and even its recurrence.

A number of obstacles still lie ahead in the study of ARD1. To use the understanding of the clinical significance of ARD1 overexpression in HCC in a clinical practice, it should be applicable for early detection (as a specific tumor marker), and curative or postoperative adjuvant treatment (as a therapeutic target). These goals are promising, given recent research findings by our group and others. In addition to other molecules (e.g., MTA1), ARD1 can be considered as a potential target in hepatocarcinogenesis and conquering HCC in the future.

## Conclusion

Since ARD1 was first identified in 1985, its potential roles have been discussed constantly. ARD1 has several isoforms and plays different roles in different species. In humans, we suggest that ARD1 has specific roles in cellular growth, differentiation, neuronal development, and cancer development/progression. In particular, ARD1 may play opposite roles—as a tumor suppressor or an oncoprotein—in the tumorigenesis of different cancer types. On the basis of our recent studies, however, ARD1 might be an oncogenic protein in hepatocarcinogenesis. Furthermore, we have revealed that ARD1 is a useful diagnostic and/or prognostic index in patients with HCC. Nonetheless, the molecular signaling pathway used by ARD1 in HCC has not been completely determined. Further investigations should continue to clarify the precise molecular mechanism of ARD1 in hepatocarcinogenesis.
